# Extended Thromboprophylaxis With Rivaroxaban, Aspirin, or No Prophylaxis After Reverse Total Shoulder Arthroplasty: Practice Patterns and 90-Day Complications in a Single-Center Retrospective Cohort Study

**DOI:** 10.7759/cureus.115992

**Published:** 2026-09-09

**Authors:** Paulo Piluski, Paula Szymanski, João Artur Bonadiman, Italo Mejía, Carlos Rodriguez, Osvandre Lech

**Affiliations:** 1 Orthopedics, Hospital São Vicente de Paulo, Instituto de Ortopedia e Traumatologia, Passo Fundo, BRA

**Keywords:** aspirin, direct oral anticoagulant, postoperative complications, pulmonary embolism, reverse shoulder arthroplasty, rivaroxaban, thromboprophylaxis, venous thromboembolism

## Abstract

Background and objective

Venous thromboembolism (VTE) after reverse total shoulder arthroplasty (RTSA) is uncommon but potentially fatal, and there is no consensus on extended pharmacological prophylaxis after discharge. This study described symptomatic VTE and other acute complications within 90 days of RTSA under the three extended prophylaxis strategies used in routine practice (rivaroxaban, aspirin, or no extended prophylaxis) and compared them descriptively.

Methods

We retrospectively reviewed all RTSAs performed by four shoulder and elbow surgeons at a single orthopedic referral center between January 2021 and April 2024. Patients with preoperative oral anticoagulation, follow-up shorter than three months, or a post-discharge regimen other than the three studied or not completed were excluded. All patients received intraoperative mechanical prophylaxis, enoxaparin 40 mg daily until discharge, and early mobilization. After discharge, in the absence of an institutional protocol, the treating surgeon chose rivaroxaban 10 mg once daily for 15 or 30 days, aspirin 81 mg once daily for 30 days, or no extended prophylaxis. Pulmonary embolism (PE) was confirmed by computed tomography pulmonary angiography. The primary outcome, symptomatic VTE, was compared with the exact Freeman-Halton test, Holm-adjusted pairwise Fisher's exact tests, and risk differences with Newcombe hybrid score 95% confidence intervals (CIs); other complications were described without inferential testing.

Results

Of 84 patients screened, 68 were included (52 women, 76.5%; median age 76 years, interquartile range 71-79), and all completed in-person follow-up at three months. No extended prophylaxis was given to 28 patients (41.2%), aspirin to 17 (25.0%), and rivaroxaban to 23 (33.8%). No symptomatic deep vein thrombosis occurred. Symptomatic PE occurred in one of 28 patients without extended prophylaxis (3.6%, 95% CI: 0.6-17.7; postoperative day 35, fatal), one of 17 receiving aspirin (5.9%, 95% CI: 1.0-27.0; day 22), and none of 23 receiving rivaroxaban (0%, 95% CI: 0-14.3) (p=0.717); no pairwise comparison approached significance after adjustment. Three non-thromboembolic complications occurred, all with rivaroxaban. Groups differed at baseline in age (p=0.019) and length of stay (p=0.019), and prophylaxis was almost entirely determined by the operating surgeon (p<0.001).

Conclusions

Because extended prophylaxis was chosen by the operating surgeon and was almost entirely collinear with surgeon identity, the observed differences cannot be attributed to the drugs themselves. No symptomatic PE occurred with rivaroxaban, but with only two events the estimates are highly imprecise and no comparison approached significance. These hypothesis-generating data describe institutional practice and do not establish the superiority of any strategy; extended thromboprophylaxis after RTSA should remain an individualized decision until adequately powered prospective studies are available.

## Introduction

Reverse total shoulder arthroplasty (RTSA) was initially developed to treat older patients with glenohumeral osteoarthritis associated with chronic rotator cuff insufficiency. As clinical outcomes improved, its indications expanded to include massive rotator cuff tears, acute fractures, fracture sequelae, inflammatory arthritis, failed anatomic shoulder arthroplasty, and tumors. This broader spectrum of indications has been accompanied by an increase in the absolute number of complications and revision procedures [1,2].

Complications after RTSA are influenced by the primary surgical indication, surgical technique, implant design, patient comorbidities, and surgeon experience [3,4]. Reported complications include instability, infection, hematoma, fractures, neurologic deficits, component-related problems, and the need for reoperation or revision [1-4].

Venous thromboembolism (VTE), including deep vein thrombosis (DVT) and pulmonary embolism (PE), has been less extensively characterized after shoulder arthroplasty than after lower-extremity arthroplasty. In a systematic review of nine studies comprising 12,566 shoulders, the pooled incidence of VTE after shoulder arthroplasty was 0.81%, with 0.54% for DVT and 0.33% for PE; mortality among patients who developed VTE reached 4.1%, and 8.3% among those with PE [5]. A review of medical complications after shoulder arthroplasty similarly reported VTE rates below 1%, with arthroplasty performed for fracture identified as the leading risk factor, alongside preoperative anemia, heart failure, chronic obstructive pulmonary disease, obesity, and increasing age [6]. Larger series have shown that symptomatic events, although rare, may be severe and occasionally fatal [7-13].

The low absolute event rate and the competing concern for postoperative bleeding or hematoma make routine pharmacological thromboprophylaxis after shoulder arthroplasty controversial, and dedicated consensus recommendations for shoulder and elbow surgery have been published to address this uncertainty [14]. Perioperative antithrombotic management also requires caution, because continuation of antiplatelet therapy such as clopidogrel may increase perioperative blood loss and complications [15]. Low-dose aspirin has been proposed as a low-bleeding-risk alternative and has been associated with low rates of symptomatic VTE after primary shoulder arthroplasty [16], whereas randomized data in hip and knee arthroplasty found aspirin to be noninferior to rivaroxaban for extended prophylaxis after an initial course of rivaroxaban [17]. A recent large matched cohort analysis found no reduction in VTE, PE, or DVT with chemical prophylaxis after total shoulder arthroplasty [18].

Most available evidence, however, treats chemoprophylaxis as a single category or addresses in-hospital prophylaxis only. Few studies describe what happens after discharge, when the surgeon must choose between an oral anticoagulant, an antiplatelet agent, or nothing at all. This study therefore described symptomatic thromboembolic events and other acute complications within 90 days after RTSA under the three extended prophylaxis strategies used in routine practice at a single center, rivaroxaban, aspirin, or no extended prophylaxis, and compared them descriptively. Because the strategy was chosen by the treating surgeon rather than allocated by protocol, the objective was to characterize institutional practice patterns and 90-day outcomes, not to estimate the comparative effectiveness of the three strategies.

## Materials and methods

Study design and ethics

This retrospective descriptive cohort study was approved by the local institutional Research Ethics Committee. Written informed consent for participation in this study was obtained from every patient. For patients operated on in 2023 and 2024, consent was obtained before surgery, as part of the preoperative documentation, so that it was documented before any postoperative outcome had occurred; this includes the patient who subsequently died. For patients operated on in 2021 and 2022, before the study was initiated, consent was obtained retrospectively and in person at a subsequent follow-up visit. In every case, consent was given by the patient; no consent was given by a representative, and none was waived. No patient declined to participate. The implications of retrospective consent for patient selection are addressed in the limitations. The report follows the Strengthening the Reporting of Observational Studies in Epidemiology (STROBE) recommendations for cohort studies [19].

Patients and surgical setting

The study population was defined as all patients who underwent RTSA between January 2021 and April 2024 at a single orthopedic referral center, and all such patients were screened for inclusion. Patients who underwent anatomic total shoulder arthroplasty or hemiarthroplasty during the same period fell outside this population and were therefore not screened. All procedures were performed by one of four shoulder and elbow surgeons from the same surgical team. Multiple implant designs were used: Implantcast (Implantcast GmbH, Buxtehude, Germany), Stryker/Tornier (Stryker Corporation, Kalamazoo, Michigan, USA), Exactech (Exactech Inc., Gainesville, Florida, USA), Zimmer Biomet (Zimmer Biomet Holdings Inc., Warsaw, Indiana, USA), and Lima (LimaCorporate S.p.A., Villanova di San Daniele del Friuli, Italy). Surgical indications included rotator cuff tear arthropathy, primary glenohumeral osteoarthritis, proximal humerus fractures and fracture sequelae, dislocations, revision of previous osteosynthesis, and conversion of previous anatomic total shoulder arthroplasty or hemiarthroplasty to RTSA.

Exclusion criteria were defined as follows: patients whose medical records could not be accessed or were incomplete for the variables of interest; patients who did not consent to participate in the study; patients with less than three months of postoperative follow-up; patients who were already receiving oral anticoagulation before surgery, a criterion that also captured all patients with a documented history of previous thromboembolic events, since these patients were anticoagulated on that indication; and patients whose extended prophylaxis after discharge used an agent or a dosing regimen other than the three strategies defined below, or who did not complete the prescribed course, so that each exposure group would be pharmacologically homogeneous and would reflect the full intended exposure.

Anesthesia and perioperative thromboprophylaxis

All patients received general anesthesia combined with an interscalene brachial plexus block. Mechanical thromboprophylaxis was applied intraoperatively, and all patients received enoxaparin 40 mg subcutaneously once daily throughout the hospital stay until the day of discharge. All patients were mobilized on the first postoperative day. In-hospital prophylaxis was therefore uniform in kind across the entire cohort, and the exposure under study was the antithrombotic strategy continued after hospital discharge.

Exposure groups

No standardized institutional protocol for extended prophylaxis was in place during the study period, and both the choice of agent and, for rivaroxaban, the duration of treatment were determined by the routine practice preference of the treating surgeon. Three groups were defined: (i) No extended prophylaxis: patients discharged without any antithrombotic drug; (ii) Aspirin: patients discharged on aspirin 81 mg once daily for 30 days. Aspirin is an antiplatelet agent rather than an anticoagulant and is analyzed separately for this reason; (iii) Rivaroxaban: patients discharged on rivaroxaban 10 mg once daily, for 15 days in nine patients and for 30 days in 14, according to surgeon preference; both durations lie within the range of extended regimens evaluated in randomized trials of lower-limb arthroplasty [17].

Because completion of the prescribed course was an eligibility requirement, every patient in the aspirin and rivaroxaban groups completed the full prescribed duration; adherence was therefore uniform within each group by design, and no included patient discontinued prophylaxis prematurely.

Data collection and outcomes

Medical records were retrospectively reviewed for demographic characteristics, preoperative diagnosis coded according to the International Classification of Diseases, Tenth Revision (ICD-10), 2019 version [20], laterality, implant used, operating surgeon, length of hospital stay, postoperative complications, and symptomatic thromboembolic events, including the postoperative day on which each event occurred. Follow-up was based on the routine schedule of the service, which includes an in-person visit at approximately three months after arthroplasty; patients who missed this visit were to be contacted by telephone, and those who could not be reached were excluded for incomplete follow-up. Complications occurring within 90 days after surgery were identified from the clinical, surgical, and readmission records of the institution and from direct questioning at the follow-up visit. Admissions to other hospitals were not captured systematically and could be identified only if reported by the patient or family at that visit.

The primary outcome was symptomatic VTE, defined as symptomatic PE or DVT, identified during the 90-day postoperative period. The secondary outcome was the occurrence of any acute postoperative complication within the same interval. Clinically suspected PE was investigated and confirmed by computed tomography pulmonary angiography, the reference diagnostic standard for acute PE recommended by the 2019 European Society of Cardiology (ESC) guidelines [21], so both events reported here were objectively documented. Symptomatic DVT was defined, in accordance with the same guidelines, as a clinically suspected event confirmed by compression ultrasonography of the affected limb [21]. No routine imaging screening for asymptomatic VTE was performed, and therefore only clinically detected events were included; investigation was symptom-driven, and the decision to request imaging was made by the treating clinicians.

Bleeding events were not defined as a study outcome and were not systematically documented in the source records; retrospective retrieval of this information was not possible. Comorbidities and body mass index were not systematically available in the retrospective dataset and therefore could not be included in the between-group analysis.

Statistical analysis

Analyses were performed using Python version 3.11 (Python Software Foundation, Wilmington, Delaware, USA) with the SciPy [22] and statsmodels [23] libraries. The distribution of continuous variables was assessed with the Shapiro-Wilk test [24]. Age was not normally distributed in the full cohort (p<0.001); continuous variables are therefore presented as median with interquartile range (IQR) and compared across the three groups with the Kruskal-Wallis test [25], with means and standard deviations also reported to allow comparison with previous series. Categorical variables are presented as counts and percentages.

Because the number of events was small and most expected cell counts were below five, categorical variables were compared across the three groups with the Freeman-Halton extension of Fisher's exact test [26]. Given the size of the contingency tables, exact p-values were obtained by complete enumeration of all tables compatible with the observed marginal totals, so no simulation-based approximation was used. The primary outcome was the only outcome tested inferentially. The three pairwise comparisons of the primary outcome were performed with the two-sided Fisher's exact test and adjusted for multiplicity with the Holm method [27]; both unadjusted and adjusted p-values are reported. The secondary outcome, any acute postoperative complication, was described by group without inferential testing, because its components (PE, seroma, dislocation, and nerve injury) have different mechanisms and only the thromboembolic and bleeding-related components have any plausible relationship with antithrombotic exposure; a formal comparison of this composite could suggest a safety signal driven by events unrelated to the drugs. Individual non-thromboembolic complications were likewise described without inferential testing because each occurred only once. Preoperative diagnoses were collapsed into degenerative indications (rotator cuff tear arthropathy and primary glenohumeral osteoarthritis) and trauma-related or revision indications for inferential comparison, because several individual categories contained fewer than five patients.

Proportions are reported with Wilson score 95% confidence intervals (CIs) [28]. Pairwise between-group differences in the primary outcome are reported as absolute risk differences in percentage points with Newcombe hybrid score 95% CIs [29], using the group without extended prophylaxis as the reference. Given the zero-event cells and the very small number of outcome events, relative risks and odds ratios would yield unstable and poorly informative estimates; absolute risk differences with 95% CIs were therefore emphasized. Statistical significance was defined as a two-sided p<0.05. Apart from the comparison of the primary outcome, all analyses are exploratory and are reported as such.

## Results

Eighty-four patients who underwent RTSA during the study period were screened. Sixteen were excluded: six were already receiving oral anticoagulation before surgery, including all patients with a previous thromboembolic event; five had incomplete medical records or could not be contacted for follow-up; and five received an antithrombotic agent or dosing regimen other than the three strategies studied, including the two patients treated with dabigatran. No patient declined to participate; patients from the earlier period who could not be reached to give retrospective consent are included among the five excluded for incomplete records or lack of contact. The remaining 68 patients met the eligibility criteria and constituted the study cohort (Figure 1). The cohort comprised 52 women (76.5%) and 16 men (23.5%), with a median age of 76 years (IQR: 71-79; mean 74.2, SD 8.3; range 41-91). The main surgical indications were rotator cuff tear arthropathy (ICD-10 M75.1) in 47 patients (69.1%), primary glenohumeral osteoarthritis (ICD-10 M19.0) in 14 (20.6%), proximal humerus fracture (ICD-10 S42.2) in four (5.9%), shoulder dislocation (ICD-10 S43.0) in two (2.9%), and mechanical complication of an internal joint prosthesis (ICD-10 T84.0) in one (1.5%). The median length of hospital stay was two days (IQR: 1-2; range 1-3). After discharge, 28 patients (41.2%) received no extended prophylaxis, 17 (25.0%) received aspirin 81 mg once daily for 30 days, and 23 (33.8%) received rivaroxaban 10 mg once daily for 15 days in nine patients and for 30 days in 14; all patients in the aspirin and rivaroxaban groups completed the prescribed course. All 68 patients attended the in-person follow-up visit at approximately three months after surgery, so no patient in the final cohort required telephone follow-up, and outcome ascertainment was complete and did not differ across prophylaxis groups or surgeons.

**Figure 1:**
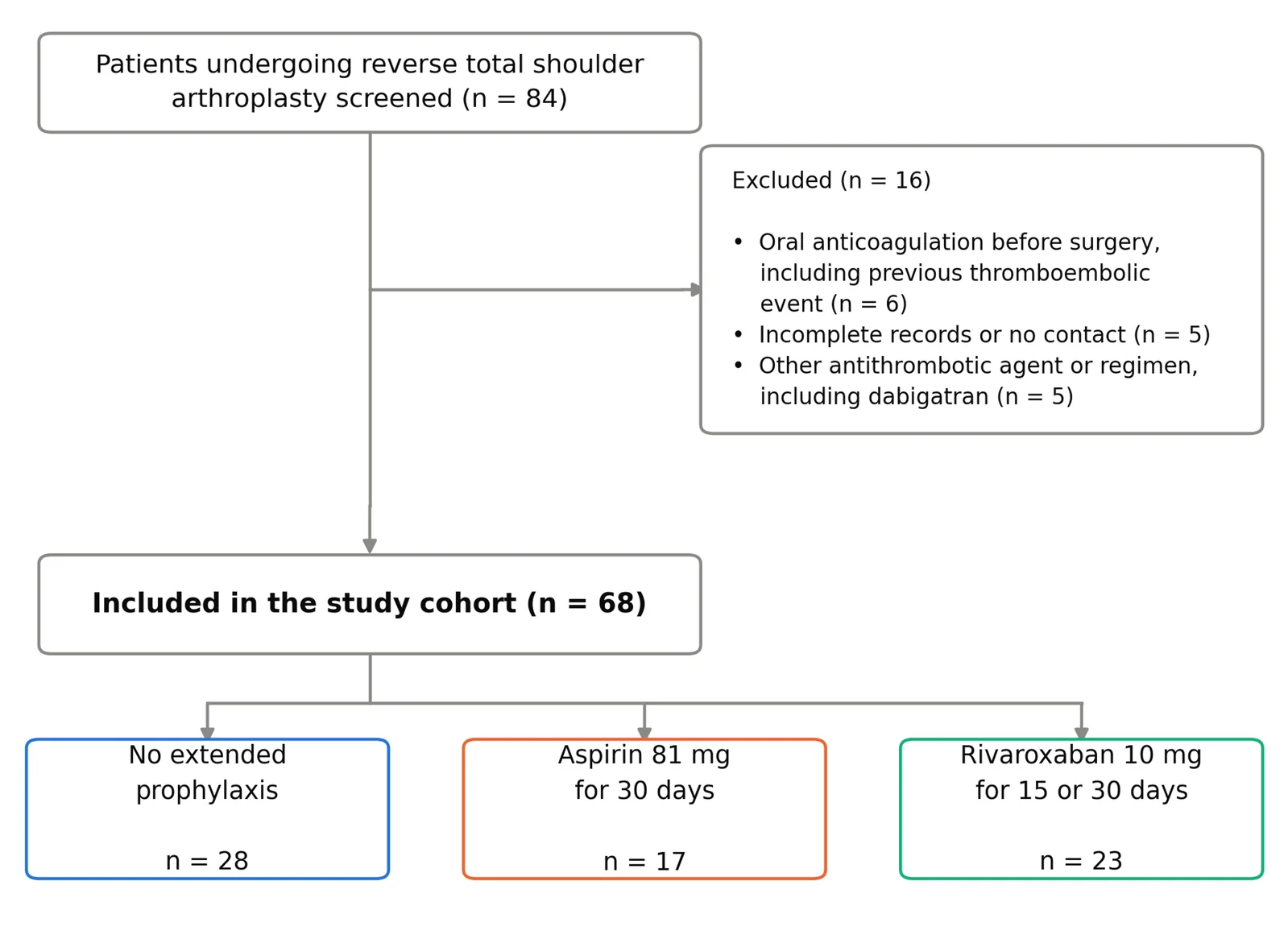
Study flow diagram showing screening, exclusions, and allocation to the three extended antithrombotic prophylaxis strategies.

Baseline characteristics according to group are presented in Table 1. Sex, laterality, and implant used did not differ significantly across groups. Age differed (median 73, 76, and 78 years in the no-prophylaxis, aspirin, and rivaroxaban groups, respectively; p=0.019), as did length of hospital stay, which was shortest in the rivaroxaban group (median two, two, and one day; p=0.019). Trauma-related or revision indications were numerically concentrated in the rivaroxaban group (7.1%, 0%, and 21.7%), and patients aged 70 years or older were less frequent in the no-prophylaxis group (67.9%, 94.1%, and 87.0%), but neither difference reached statistical significance (p=0.067 and p=0.080, respectively).

**Table 1:** Baseline characteristics according to extended antithrombotic prophylaxis after hospital discharge. Continuous variables were compared with the Kruskal-Wallis test and categorical variables with the Freeman-Halton extension of Fisher's exact test, computed exactly by complete enumeration. A single hyphen (-) indicates a cell for which no value applies. Degenerative indications comprise rotator cuff tear arthropathy and primary glenohumeral osteoarthritis; trauma-related or revision indications comprise proximal humerus fracture, shoulder dislocation, and mechanical complication of a previous prosthesis. Surgeons are labeled A to D to preserve anonymity; surgeons A and B treated 14 and five patients in total, surgeon C treated 18, and surgeon D treated 31. Comorbidities and body mass index were not systematically available in the retrospective dataset and could not be included in this comparison. IQR: interquartile range; SD: standard deviation

Characteristic	No extended prophylaxis (n=28)	Aspirin (n=17)	Rivaroxaban (n=23)	p-value
Age, years, median (IQR)	73 (68-77)	76 (75-81)	78 (72-81)	0.019
Age, years, mean ± SD	71.7±7.7	76.1±10.5	76.0±6.5	-
Age ≥70 years, n (%)	19 (67.9)	16 (94.1)	20 (87.0)	0.080
Female sex, n (%)	19 (67.9)	13 (76.5)	20 (87.0)	0.276
Right side, n (%)	17 (60.7)	9 (52.9)	16 (69.6)	0.566
Indication, n (%)	-	-	-	0.067
Degenerative	26 (92.9)	17 (100)	18 (78.3)	-
Trauma-related or revision	2 (7.1)	0 (0)	5 (21.7)	-
Implant, n (%)	-	-	-	0.181
Implantcast	12 (42.9)	11 (64.7)	16 (69.6)	-
Stryker/Tornier	8 (28.6)	1 (5.9)	3 (13.0)	-
Exactech	4 (14.3)	5 (29.4)	2 (8.7)	-
Zimmer Biomet	3 (10.7)	0 (0)	2 (8.7)	-
Lima	1 (3.6)	0 (0)	0 (0)	-
Operating surgeon, n (%)	-	-	-	<0.001
Surgeon A	0 (0)	0 (0)	14 (60.9)	-
Surgeon B	0 (0)	0 (0)	5 (21.7)	-
Surgeon C	0 (0)	17 (100)	1 (4.3)	-
Surgeon D	28 (100)	0 (0)	3 (13.0)	-
Length of hospital stay, days, median (IQR)	2 (2-2)	2 (1-2)	1 (1-2)	0.019

Prophylaxis strategy was almost entirely determined by the operating surgeon (p<0.001). All 28 patients without extended prophylaxis were operated on by a single surgeon, who treated 31 patients in total, and all 17 patients receiving aspirin were operated on by a second surgeon, who treated 18 patients in total. The rivaroxaban group comprised all 14 patients of a third surgeon, all five patients of a fourth surgeon, three patients of the first surgeon, and one patient of the second. Group membership and operating surgeon were therefore nearly collinear.

Five patients (7.4%, 95% CI: 3.2-16.1) experienced an acute complication within the first 90 postoperative days: PE in two patients (2.9%, 95% CI: 0.8-10.1), seroma in one (1.5%), dislocation in one (1.5%), and radial nerve neuropraxia in one (1.5%). No symptomatic DVT was documented, so the primary outcome of symptomatic VTE consisted entirely of the two PE events, and all comparisons of the primary outcome reported below refer to symptomatic PE. The remaining 63 patients (92.6%) had no acute complication.

Both PE events were confirmed by computed tomography pulmonary angiography, and both occurred after hospital discharge and after the end of in-hospital enoxaparin. Symptomatic PE occurred in one of 28 patients without extended prophylaxis (3.6%, 95% CI: 0.6-17.7), one of 17 patients receiving aspirin (5.9%, 95% CI: 1.0-27.0), and none of 23 patients receiving rivaroxaban (0%, 95% CI: 0-14.3). The difference across groups was not significant (p=0.717). Compared with no extended prophylaxis, the absolute risk difference was +2.3 percentage points for aspirin (95% CI: -12.6 to +23.6; unadjusted p=1.000) and -3.6 percentage points for rivaroxaban (95% CI: -17.7 to +11.0; unadjusted p=1.000); the comparison between rivaroxaban and aspirin yielded a difference of -5.9 percentage points (95% CI: -27.0 to +9.2; unadjusted p=0.425). All Holm-adjusted p-values were 1.000. The PE in the aspirin group occurred on postoperative day 22, during the prescribed 30-day course of aspirin, and the patient survived. The PE in the no-prophylaxis group occurred on postoperative day 35, more than four weeks after discharge, in a patient under 50 years of age who had undergone surgery for a trauma-related or revision indication; the event was fatal, and death was attributed to massive PE confirmed by computed tomography pulmonary angiography.

Any acute complication occurred in one of 28 patients without extended prophylaxis (3.6%), one of 17 receiving aspirin (5.9%), and three of 23 receiving rivaroxaban (13.0%); this composite was described without inferential testing, as specified in the statistical analysis plan. The seroma, dislocation, and radial nerve neuropraxia all occurred in the rivaroxaban group, and all three affected patients of the same surgeon. Complications by group are summarized in Table 2 and Figure 2.

**Table 2:** Acute postoperative complications within 90 days according to extended antithrombotic prophylaxis after hospital discharge. Values are presented as n (%). The p-value is from the Freeman-Halton extension of Fisher's exact test across the three groups, computed exactly by complete enumeration, and was calculated for the primary outcome only; because no symptomatic deep vein thrombosis occurred, the primary outcome of symptomatic venous thromboembolism coincides with the pulmonary embolism row. A single hyphen (-) indicates an outcome that was not tested inferentially: the composite of any acute complication was described without inferential testing because its components have different mechanisms and only some of them have a plausible relationship with antithrombotic exposure, and individual non-thromboembolic events each occurred only once. Pairwise comparisons, absolute risk differences, and 95% confidence intervals for symptomatic pulmonary embolism are reported in the text. The reported death occurred in one of the patients with pulmonary embolism and does not represent an additional complication.

Outcome	No extended prophylaxis (n=28)	Aspirin (n=17)	Rivaroxaban (n=23)	Total (n=68)	p-value
Symptomatic pulmonary embolism	1 (3.6)	1 (5.9)	0 (0)	2 (2.9)	0.717
Symptomatic deep vein thrombosis	0 (0)	0 (0)	0 (0)	0 (0)	-
Seroma	0 (0)	0 (0)	1 (4.3)	1 (1.5)	-
Dislocation	0 (0)	0 (0)	1 (4.3)	1 (1.5)	-
Radial nerve neuropraxia	0 (0)	0 (0)	1 (4.3)	1 (1.5)	-
Any acute complication	1 (3.6)	1 (5.9)	3 (13.0)	5 (7.4)	-
No acute complication	27 (96.4)	16 (94.1)	20 (87.0)	63 (92.6)	-
Death	1 (3.6)	0 (0)	0 (0)	1 (1.5)	-

**Figure 2:**
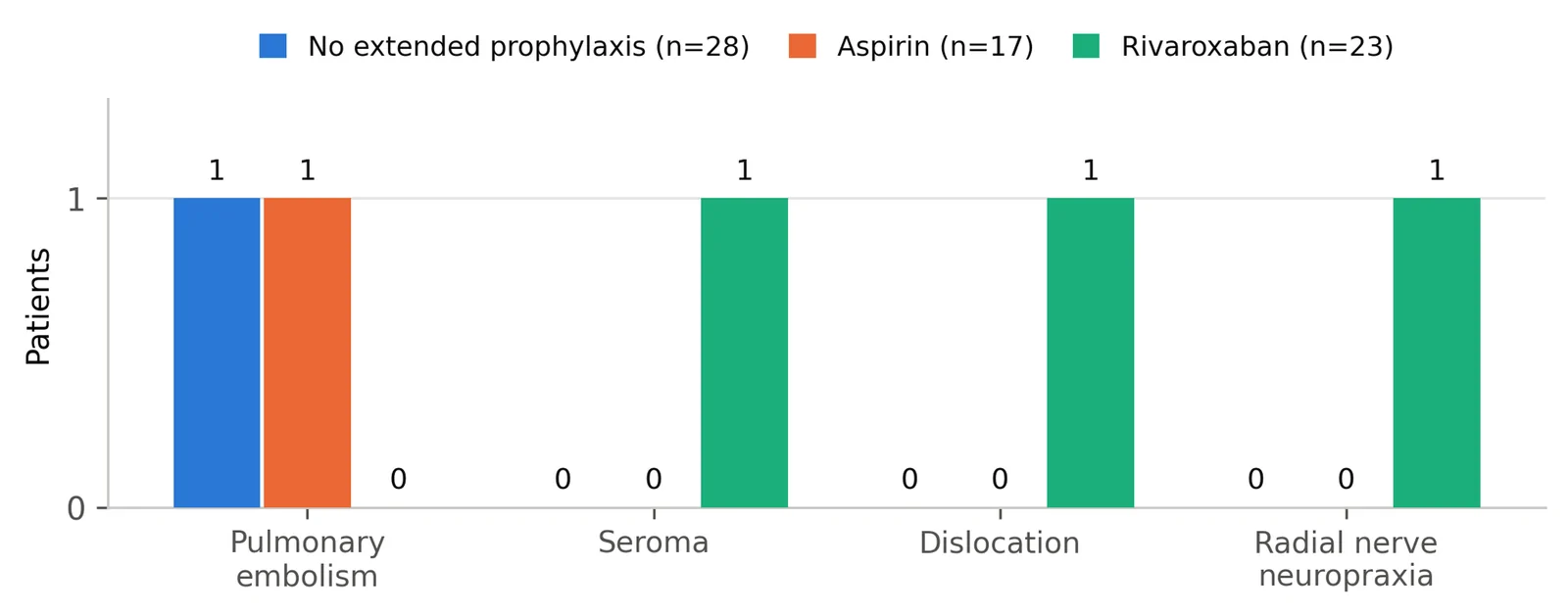
Acute postoperative complications within 90 days after reverse total shoulder arthroplasty according to extended antithrombotic prophylaxis strategy. Patients without acute complications are not shown.

## Discussion

The principal observation of this study was that no symptomatic PE occurred among the 23 patients who received rivaroxaban after discharge, whereas one event occurred among the 28 patients who received no extended prophylaxis and one among the 17 who received aspirin. None of these differences approached statistical significance, either across the three groups or in any pairwise comparison, and every CI was wide enough to include both clinically important benefit and clinically important harm. With two events in the entire cohort, this is the expected result rather than a surprising one. The absence of PE in the rivaroxaban group is compatible with a true event rate of up to 14.3%, which is the upper confidence limit for zero events in 23 patients; that limit is more than an order of magnitude above the incidence reported in large series, which illustrates how little this sample constrains the underlying risk.

The most important limitation of this cohort is not its size but its structure. Prophylaxis strategy and operating surgeon were almost entirely collinear: every patient in the no-prophylaxis group was treated by one surgeon, and every patient in the aspirin group was treated by another. Consequently, comparisons among prophylaxis groups are strongly confounded by surgeon-specific practice patterns, and any difference between the groups could equally reflect differences in surgical technique, implant preference, case selection, perioperative care, threshold for investigating symptoms, or intensity of follow-up. This is not a residual concern that statistical adjustment could address: with 68 patients, five events, and near-complete separation between surgeon and exposure, surgeon effects and drug effects are not identifiable in these data. We report the comparison because it describes real institutional practice and because concealing the structure would misrepresent the study, but no causal inference about the drugs themselves is supportable. The observation that all three non-thromboembolic complications in the rivaroxaban group occurred in patients of a single surgeon illustrates the same point.

Baseline characteristics also differed across groups in a way that is consistent with how surgeons choose prophylaxis: the rivaroxaban group had an older age distribution, a numerically higher proportion of trauma-related or revision indications, and the shortest hospital stay. Advanced age and traumatic or revision indications are recognized risk factors for VTE after shoulder arthroplasty [5,6]. This is a descriptive observation only. Comorbidities, body mass index, operative time, blood loss, mobility, and thromboembolic risk scores were not available; the imbalance in indication did not reach statistical significance; and unmeasured differences between surgeons remain a sufficient explanation for any pattern in the data, so no conclusion about the baseline thromboembolic risk of the rivaroxaban group relative to the other two groups can be drawn.

The overall symptomatic PE rate in this cohort was 2.9%, with a 95% CI of 0.8-10.1%. Previous studies have reported considerably lower rates. In a statewide database of 13,755 shoulder arthroplasties, Lyman et al. reported a DVT rate of 5.0 per 1,000 procedures (0.50%) and a PE rate of 2.3 per 1,000 procedures (0.23%) [9]. Sperling and Cofield identified five pulmonary emboli among 2,885 consecutive primary shoulder arthroplasties, a prevalence of 0.17%, none of which was fatal, and noted that in three of the five patients the initial symptoms were attributed to other causes [10]. Kolz et al. reported updated rates of symptomatic thromboembolic complications from a large institutional series [13], and Navarro et al. reported symptomatic VTE in 1.01% of 2,574 shoulder arthroplasties, comprising 0.51% DVT and 0.54% PE [12]. Saleh et al. and Na et al. demonstrated substantial variability across the literature, reflecting differences in study design, diagnostic criteria, patient selection, and surveillance methods [5,11]. The point estimate observed here is therefore higher than most published figures, but its CI overlaps them, and the apparent difference is most plausibly explained by the small denominator, in which a single additional event shifts the rate by 1.5 percentage points.

The clinical relevance of VTE after shoulder surgery remains substantial despite its low absolute incidence, because PE can be fatal; VTE-related mortality of approximately 4% and PE-related mortality of approximately 8% have been reported [5]. In the present cohort, one of the two patients with PE died. The fatal event occurred on postoperative day 35, more than four weeks after discharge and well after the end of in-hospital enoxaparin, in a patient under 50 years of age who had undergone surgery for a trauma-related or revision indication, a setting associated with increased thromboembolic risk. Trauma-related indications may likewise represent a particularly relevant context: Heyer et al. specifically evaluated VTE after surgical treatment of proximal humerus fractures [7], Navarro et al. observed a trend toward more frequent VTE after shoulder arthroplasty performed for traumatic rather than elective indications (1.71% versus 0.80%; p=0.055) [12], and King et al. identified arthroplasty for fracture as the leading risk factor for VTE [6]. These observations argue for individualized risk assessment rather than for assumptions of uniformly low risk based on age or indication alone, and the timing of both events in this cohort, on postoperative days 22 and 35, is a reminder that the period of thromboembolic risk after RTSA extends well beyond the hospital stay.

Analyzing aspirin separately, rather than treating every patient without an anticoagulant as unexposed, was a deliberate choice. Aspirin is an antiplatelet agent with a different mechanism and a different bleeding profile. It has been associated with low rates of symptomatic VTE after primary shoulder arthroplasty [16], and it was noninferior to rivaroxaban for extended prophylaxis after hip and knee arthroplasty in a randomized trial [17]. That trial, however, evaluated aspirin only after an initial five-day course of rivaroxaban in lower-limb arthroplasty, a regimen that differs materially from aspirin monotherapy after shoulder arthroplasty, so it provides biological and clinical context rather than direct evidence for the regimen studied here. In the present cohort, the aspirin group had the numerically highest PE rate, at 5.9%, but this represents a single event in 17 patients and is uninformative in isolation; the event occurred on postoperative day 22, while the patient was still taking aspirin, so it was a breakthrough event during prophylaxis rather than an event after its completion. Grouping aspirin with no prophylaxis, as earlier analyses of this cohort did, would have obscured a pharmacologically meaningful distinction without adding interpretable information.

Three non-thromboembolic complications occurred in the rivaroxaban group: one seroma, one dislocation, and one radial nerve neuropraxia. The dislocation and the neurologic event have no evident mechanistic relationship with anticoagulant exposure, and all three events occurred in patients of a single surgeon. For these reasons, the composite of any acute complication was described without inferential testing: a formal comparison would have suggested a safety disadvantage for rivaroxaban driven mainly by surgical events unrelated to the drug. The seroma is potentially relevant when considering the balance between thrombosis prevention and postoperative bleeding or fluid collection, but a single event cannot establish an association. Beyond this seroma, bleeding and wound-related events were not defined as study outcomes, were not systematically documented in the source records, and could not be retrieved retrospectively. This is a meaningful gap, because the principal argument against routine chemoprophylaxis in shoulder arthroplasty is bleeding rather than lack of efficacy, and the present data therefore speak only to the efficacy side of the risk-benefit balance.

Taken together with the published literature, these findings are consistent with the prevailing view that routine, uniform extended chemoprophylaxis is not clearly justified after shoulder arthroplasty and that risk stratification is preferable. A recent matched cohort analysis of nearly 20,000 patients found no reduction in VTE, PE, or DVT with chemical prophylaxis after total shoulder arthroplasty [18], and a review of medical complications recommended mechanical prophylaxis combined with aspirin for most patients, reserving other pharmacological agents for those identified as high risk [6]. Dedicated consensus recommendations for shoulder and elbow surgery have also been developed to guide these decisions [14].

This study has strengths, including consecutive evaluation of patients from a single referral center, a uniform in-hospital prophylaxis and anesthesia protocol across the whole cohort, a defined 90-day postoperative observation period with in-person follow-up completed by all 68 patients, objective confirmation of both thromboembolic events by computed tomography pulmonary angiography rather than clinical diagnosis alone, documentation of the timing of both events relative to discharge and to the prescribed prophylaxis, the separation of antiplatelet from anticoagulant exposure rather than their conflation, and reporting of effect estimates with CIs rather than p-values alone for the primary outcome.

Several limitations should be acknowledged. The retrospective design introduces the possibility of information bias and underreporting. The sample size was modest; the three groups contained 28, 17, and 23 patients, and only two symptomatic thromboembolic events occurred, so the uncertainty around every estimate is wide. As discussed above, prophylaxis strategy was almost entirely clustered by surgeon, so drug effects and surgeon effects cannot be separated. Comorbidities and body mass index were not systematically available in the retrospective dataset, nor were previous thromboembolic risk scores, operative time, and intraoperative blood loss. This is a substantive limitation rather than an incidental one, because obesity, diabetes, cardiac arrhythmia, heart failure, chronic obstructive pulmonary disease, and preoperative anemia are among the established risk factors for VTE after shoulder arthroplasty [5,6]; baseline comparability could therefore be assessed only for the variables shown in Table 1, and the possibility that the groups differed in unmeasured thromboembolic risk cannot be excluded in either direction.

A further and less obvious confounder concerns in-hospital prophylaxis. Although every patient received enoxaparin 40 mg once daily, this was given only until discharge, and length of stay differed significantly across groups. Patients in the rivaroxaban group were discharged a median of one day earlier and therefore received, on average, a shorter course of parenteral prophylaxis than the other two groups. Total antithrombotic exposure was consequently not simply a function of the discharge prescription, and the groups differed in the perioperative component of prophylaxis as well as in the extended component.

Additional limitations include the lack of standardization of implant selection and surgical technique; symptom-driven ascertainment of VTE, so that differences between clinicians in the threshold for requesting imaging could have produced surveillance bias, although the in-person follow-up visit was completed by every patient and did not differ by group; the possibility that admissions to other hospitals were missed unless reported by the patient at that visit; the recording of clinically detected events only, so that asymptomatic VTE may have been missed; the absence of bleeding as a predefined outcome; the variable duration of rivaroxaban, which ranged from 15 to 30 days according to surgeon preference and which the sample is far too small to compare; the requirement that patients complete the prescribed course, which makes the aspirin and rivaroxaban groups per-protocol populations from which any patient who stopped prophylaxis early, for example, because of bleeding or intolerance, would have been excluded; the exclusion of two patients treated with dabigatran, which improved pharmacological homogeneity but slightly reduced the sample; and the single-center design, which limits generalizability.

The timing of consent also deserves comment. For patients operated on in 2021 and 2022, consent was obtained retrospectively at an in-person follow-up visit, so a patient from that period who had died or was too unwell to attend before consent could be obtained would not have been included; outcome-dependent selection bias therefore cannot be excluded for that part of the cohort, and the number of events in it may be underestimated. For patients operated on from 2023 onward, including the patient who died, consent was obtained before surgery and could not have been influenced by outcome.

These findings should therefore be viewed as descriptive and hypothesis-generating. Larger multicenter prospective studies, with prophylaxis allocated by protocol rather than by surgeon preference, standardized definitions, systematic assessment of both thromboembolic and bleeding outcomes, and adequate adjustment for patient- and procedure-related risk factors, are needed to determine whether extended pharmacological thromboprophylaxis provides a net clinical benefit after RTSA, and if so, in which patients and with which agent.

## Conclusions

The decisive constraint on the interpretation of this single-center cohort is structural rather than numerical: extended thromboprophylaxis after RTSA was chosen by the operating surgeon and was almost entirely collinear with surgeon identity, so the observed differences cannot be attributed to the drugs themselves. Within that constraint, symptomatic PE was uncommon but clinically important, occurring weeks after discharge and, in one case, proving fatal. No PE occurred with rivaroxaban, but no comparison approached statistical significance, and with so few events the data cannot exclude a clinically relevant effect in either direction. Non-thromboembolic complications occurred only in the rivaroxaban group, all in patients of a single surgeon, and were described without inferential testing.

This study describes how extended thromboprophylaxis was practiced at one referral center and what happened to those patients; it does not establish that any strategy is superior. Extended thromboprophylaxis after RTSA should be guided by individualized assessment of thromboembolic and bleeding risk, recognizing that the period of risk extends beyond the hospital stay. Adequately powered prospective studies with protocol-driven allocation and systematic capture of efficacy and safety outcomes are required to identify which patients benefit and from which agent.
